# Supplementary figures and images for: Colletotrichum gloeosporioides Swiftly Manipulates the Transcriptional Regulation in Citrus sinensis During the Early Infection Stage
Source: J Fungi (Basel). 2024 Nov 20;10(11):805. doi: 10.3390/jof10110805 (PMC11595579; doi:10.3390/jof10110805)

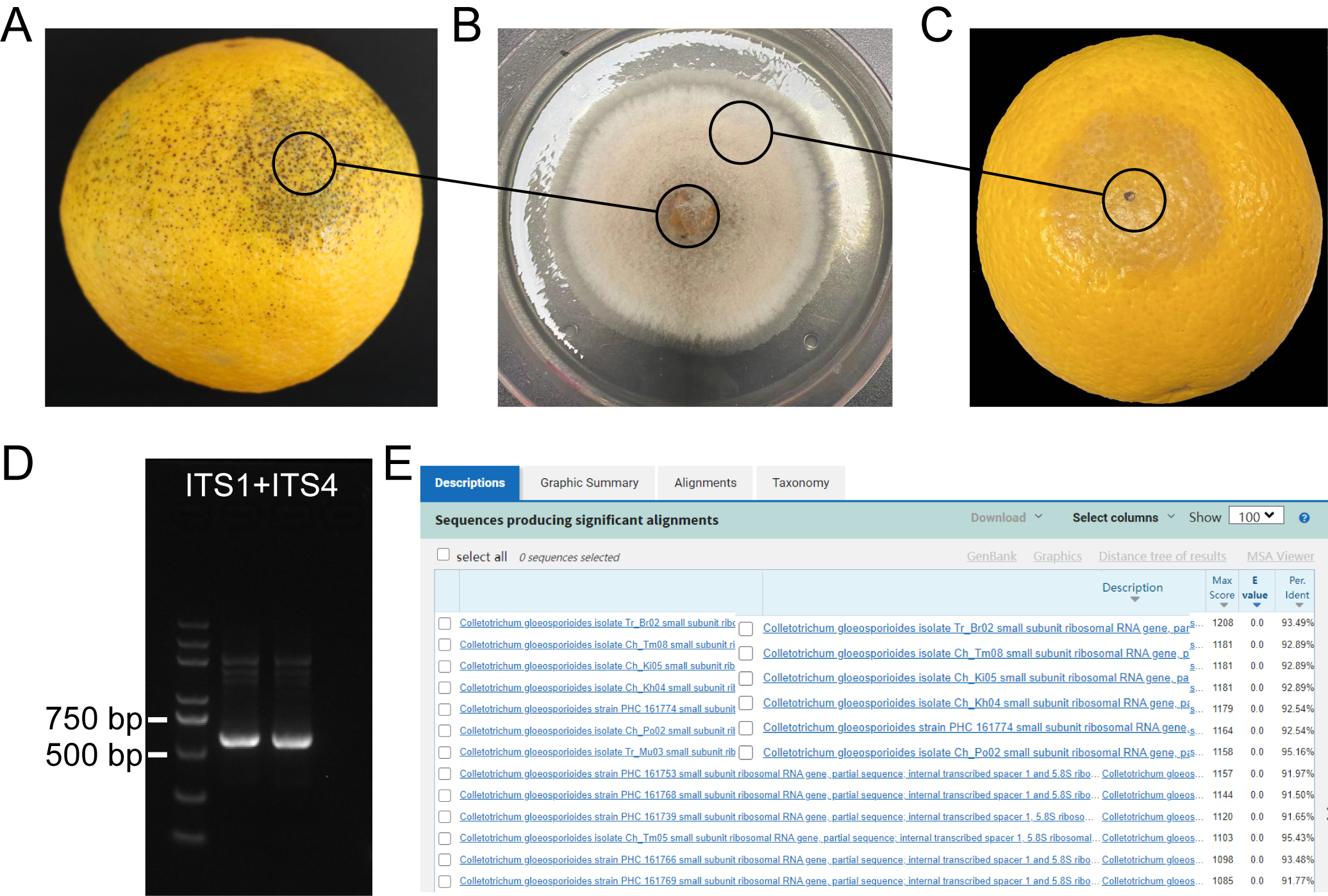

Supplement: Supplementary file 1 [file jof-10-00805-s001.zip › Figure S1.tif]

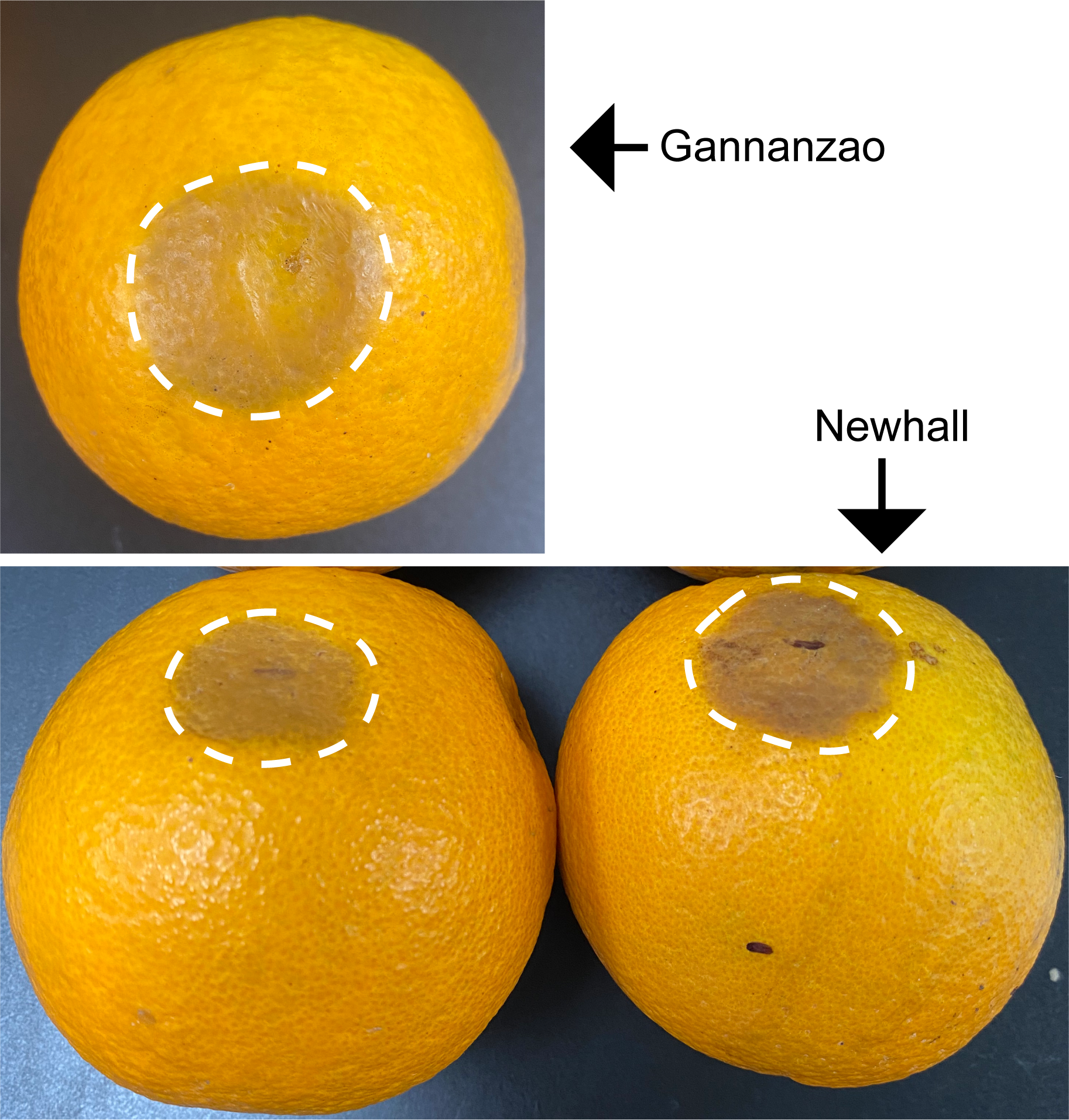

Supplement: Supplementary file 1 [file jof-10-00805-s001.zip › Figure S2.tif]
